# Determination of complex permittivity of a thin low-loss dielectric sample using state-space approach from waveguide measurements

**DOI:** 10.1038/s41598-026-49374-6

**Published:** 2026-05-25

**Authors:** Ugur C. Hasar, Husain Ali, Yunus Kaya

**Affiliations:** 1 Department of Electrical and Electronics Engineering, Gaziantep University, 27310 Gaziantep, Türkiye; 2 Applied Electromagnetics Research Laboratory (Aer-Lab), Gaziantep University, 27310 Gaziantep, Türkiye; 3 Department of Electronics and Automation, Bayburt University, 69010 Bayburt, Türkiye; 4 Department of Electrical and Electronics Engineering, Atatürk University, 25240 Erzurum, Türkiye

**Keywords:** Engineering, Materials science, Optics and photonics, Physics

## Abstract

An extraction procedure is proposed for relative complex permittivity ($\varepsilon _{rs}$) determination of thin low-loss samples positioned/deposited on a supporting substrate material using waveguide non-resonant microwave measurements. Its substrate thickness and sample thickness independence makes it unique extraction method in comparison with the available retrieval methods which are free from either substrate thickness information only or sample thickness information only. Its theoretical model is constructed on the state-transition matrix and state vector through which the link between $\varepsilon _{rs}$ and measured scattering (S-) parameters is established. Compared with previous works in the literature, a closed form (explicit) expression of $\varepsilon _{rs}$ is derived for a two-layer (sample on a substrate material) composite structure, thereby eliminating any numerical analysis or tool. Numerical analyses are implemented for validation of the proposed method as well as assessing the effect of noise and any possible air gap between the sample and its substrate. X-band (*8.2-12.4* GHz) waveguide measurements of a thinner (around 1.00 mm) polyvinyl chloride (PVC) for different measurement scenarios and measurements of a thin zinc oxide film ($\cong 1.0 \mu$m) were performed to evaluate the performance of the proposed extraction method against other similar methods in the literature. Our method can find applications for $\varepsilon _{rs}$ characterization of thin-film, radar-absorbent material coatings, anti-corrosive paints, or thermal barrier coatings where sample thickness (and substrate thickness) can vary from one sample to another from the same batch.

## Introduction

Determining of electromagnetic properties of materials are required for understanding their behavior and modeling their reflection and transmission properties^1–3^. This is critical especially for new materials with unique properties not known a priori. There are many application areas that such a determination gets benefit involving electromagnetic stealth^4^, civil engineering^5^, and agriculture^6^. Measurement methods at microwave frequencies can be categorized into resonance type methods and non-resonance type methods^7^. Resonance type microwave methods have the highest accuracy and sensitivity due to localization of electromagnetic signals within a narrow region over which the sample under analysis is present. Nevertheless, they are in general narrow-band and require meticulous sample preparation (workload). Non-resonance type microwave methods can mitigate these limitations by allowing broadband electromagnetic material characterization and requiring relatively less sample preparation. Due to ever-growing recent interest in broadband material characterization, non-resonance type microwave methods are within our current research interest.

There are plenty of non-resonance type microwave methods proposed in the literature including free-space measurements^1,3,8,9^, planar-structure measurements^10–12^, open-ended waveguide, coupled-waveguide, or coaxial probe measurements^13–17^, and conventional coaxial/waveguide line measurements^18–37^. Our main focus in the present study is the material characterization by means of conventional waveguide/coaxial line measurements due to their wider availability, broader applicability, and relatively higher accuracy.

Samples with distinct physical, chemical, and mechanical properties are composed or cascaded to construct a composite structure in order to harness different operational behavior and improve functionality. Electromagnetic composite structures find applications in different diverse areas including, to name a few, electromagnetic absorbtion^38^, defect/damage diagnosis^39^, and electromagnetic shielding or interference^40^. Many engineered composites or specialized polymers are fabricated in the form of thin layers in cascade manner. This circumstance poses a challenge for electromagnetic characterization of these individual layers as well as the overall structure, because overall electromagnetic response of a composite structure cannot be simply modeled or predicted with electromagnetic constitutive parameters of individual layers due to an established interference pattern over the entire structure (interfacial and thickness effect) due to passage of electromagnetic signals through thin transparent adjacent individual layers. It is therefore a need for electromagnetic parameter retrieval of composite structures.

The retrieval methods in the studies based on waveguide/coaxial line measurements^2,18–26,37^ are applicable for one-layer medium only and thus are not implementable for electromagnetic parameter retrieval of composite structures. For a two-layer electromagnetic property analysis, the methods in the studies^1,23,27–36^ can be utilized. While the methods in the studies^27–34^ require precise information about sample thickness and substrate thickness and permittivity for accurate electromagnetic parameter extraction of the sample, the method in the study^35^ suffers from substrate thickness and permittivity, and the method in the study^1^ is affected by sample thickness to achieve the same goal. However, improper information of sample thickness and substrate thickness will hinder accurate retrieval of electromagnetic properties due to the aforementioned interference effect. Any method free from thickness information will get benefit from accurate modeling of the composite structure. Our recent retrieval method in the study^36^ takes advantage of this circumstance in retrieving electromagnetic properties of a sample on a substrate material. However, the first reflection terms in the froward and backward directions required to implement this methodology need to be isolated from overall reflection terms in those directions. This situation hinders widespread implementation of this method because such an isolation is merely comfortably exercised either by broadband measurements or for thicker samples and substrates (or both). Additionally, such an isolation is implemented by the time-domain gating with a window having an imperfect frequency transition at the expense of inevitable ripples around the initial and final frequencies of the re-transformed spectral signal^1,3^. To eliminate these ripples in the extracted electromagnetic properties, a retrieval method implemented directly with spectral measured information only is a necessity. In this study, we propose a retrieval method free-from substrate thickness and sample thickness information for accurate retrieval complex permittivity of a sample on a substrate material using spectral scattering (S-) parameter measurements only (no need for time-domain gating procedure). A comparison of various non-resonance type transmission-reflection retrieval techniques^1–3,10–12,27–36^ is presented in Table 1.

**Table 1:** Comparison of different non-resonance type retrieval techniques^1–3,10–12,27–37^.

Method	Environ.	Sample Thickness	Substrate Thickness	Limitation
Xue-1^1^	Free-Space	Required	Not Required	Isolation of first reflection/transmission terms by time-domain gating
Luo *et. al*^3^	Free-Space	Not Required	N/A	Mitigation of adverse/stray signals by means of time-domain gating
Xue-2^8^	Free-Space	Required	N/A	Mitigation of adverse/stray signals by means of time-domain gating
Xue-3^9^	Free-Space	Not Required	Required	Mitigation of adverse/stray signals by means of time-domain gating
Cai *et. al.*^10^	Planar	Required	N/A	Fabrication of two identical lines with different lengths
Chung *et. al.*^11^	Planar	Required	N/A	Fabrication of two identical lines with different lengths
Omam *et. al.*^12^	Planar	Required	N/A	Masking of high noise level with actual low-level measured data
Liu *et. al*^2^	Coaxial/WG	Required	N/A	Precise sample movement of the sample in the cell
Williams *et. al.*^23^	Coaxial/WG	Required	Required	Influence of improper substrate permittivity and thickness information
Hasar-1^27^	Coaxial/WG	Required	Required	Influence of improper substrate permittivity and thickness information
Hasar-2^28^	Coaxial/WG	Required	Required	Influence of improper substrate permittivity and thickness information
Chao *et al.*^29^	Coaxial/WG	Required	Required	Influence of improper substrate permittivity and thickness information
Hasar-Bute^30^	Coaxial/WG	Required	Required	Influence of improper substrate permittivity and thickness
Hasar-Kaya^31^	Coaxial/WG	Required	Required	Information and sample thickness information
Yang-Ma^32^	Coaxial/WG	Required	Required	
Hasar-1 et al.^33^	Coaxial/WG	Required	Required	
Hasar-Korkmaz^34^	Coaxial/WG	Required	Required	
Hasar-2 et al.^35^	Coaxial/WG	Not Required	Required	Influence of improper substrate permittivity and thickness information
Hasar-Ozturk^36^	Coaxial/WG	Not Required	Not Required	Influence of improper substrate permittivity information only and isolation of first reflection terms by the time-domain gating
Fu *et al.*^37^	Coaxial/WG	Required	N/A	Identical error boxes, applicability to non-dispersive samples, and a need for the sample thickness information
Proposed Work	Coaxial/WG	Not Required	Not Required	Influence of improper substrate permittivity information only

**Table 2:** Comparison of different non-resonance type retrieval techniques relying on the state-transition matrix.

Method	Medium	Implementation
^41,42^	One-Layer (Chiral)	Requires a numerical tool
^43^	One-Layer (Bianisotropic)	Requires a numerical tool
^44^	Inhomogeneous (Bianisotropic)	Requires an optimization
^45^	Inhomogeneous (Isotropic)	Requires a numerical tool
^46,47^	One-Layer (Bianisotropic)	Exact Solution
This Work	Two-Layer (Isotropic)	Exact Solution

On the other hand, different from previous non-resonance type waveguide/coaxial line retrieval methods^27–36^, a theoretical model established over the state-transition matrix, which relies on direct computation of the transfer matrix of a sample without requiring the application of wave impedance (as different from other retrieval methods based on S-parameters), has been applied for the first time in the literature to determine complex permittivity of the sample on a substrate material from closed-form expressions without resorting to any numerical or optimization tool. This itself renders to our original contribution on already available extraction methods in the literature^41–47^. The remaining part of our paper is structured in this way. First, Sect. 2 includes three subsections involving i) a the theoretical model over which our extraction method is established using the state transition matrix along with state vector in Subsect. 2.1, ii) an extraction procedure using close-form expressions for the permittivity by means of measured S-parameters in Subsect. 2.2, and iii) results of numerical analysis performed for validation of our proposed method and evaluating its accuracy against noise and any potential air gap present between the sample and its substrate in Subsect. 2.3. Next, Sect. 3 presents X-band (8.2 - 12.4 GHz) waveguide measurement results of a thinner polyvinyl chloride (PVC) sample positioned on its substrate material for different measurement scenarios as well as measurements of a thin film (with a thickness of around 1*μ*m). Finally, Sect. 4 summarizes all important results relevant our extraction method (Table2).

## Materials and methods

### Theoretical analysis

**Fig. 1:**
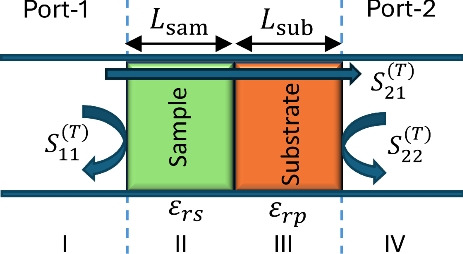
A schematic view of the sample with length $L_{\text {sam}}$ on a substrate material with length $L_{\text {sub}}$ positioned into a rectangular waveguide cell.

Fig. 1 illustrates a schematic view of waveguide section/cell involving a dielectric sample with length $L_{\text {sam}}$ positioned/fabricated on a dielectric substrate with length $L_{\text {sub}}$. Our goal is to evaluate the relative complex permittivity of the sample $\varepsilon _{rs} = \varepsilon _{rs}^{\prime } - j \varepsilon _{rs}^{\prime \prime }$ from measured forward and backward reflection and transmission S-parameters $S_{11}^{\text {(T)}}$, $S_{21}^{\text {(T)}}$, and $S_{22}^{\text {(T)}}$ given that $L_{\text {sub}}$ and the relative complex permittivity of the substrate $\varepsilon _{rp}$ are known a priori. In our theoretical analysis, state matrices are first derived. Next, relation between these matrices with measured S-parameter is established. Then, extraction procedure utilizing measured S-parameters is presented. After, this extraction procedure is validated and its accuracy is examined by a numerical analysis.

#### Determination of state matrices

Determination of the transfer (*[Γ ]*) matrix of the two-layer configuration starts with evaluation of the *[Γ ]* matrix of an individual layer. Assuming that only the dominant (TE$_{10}$) mode exists in a rectangular waveguide section in the sample region only, the following relation between the state vector *[Φ ]* and $[\Gamma _{\text {II}}]$ matrix is derived using Maxwell’s equations for a wave propagation along the *z* direction^48^

1 $$\begin{aligned} \frac{d}{d z} [\Phi (x,z)] = [\Gamma _{\text {II}}] \cdot [\Phi (x,z)] \end{aligned}$$

where

2 $$\begin{aligned} {[}\Phi ] = [0, E_y (x,z), H_x(x,z), 0]^{T}, \end{aligned}$$

3 $$\begin{aligned} {[}\Gamma _{\text {II}}] = \begin{bmatrix} 0 & 0 & 0 & 0 \\ 0 & 0 & h_{\text {II}} & 0 \\ 0 & g_{\text {II}} & 0 & 0 \\ 0 & 0 & 0 & 0 \end{bmatrix}, \ \ \ \ \begin{matrix} g_{\text {II}} = j \dfrac{ (k_0^2 \varepsilon _{rs} - k_x^2) }{ \omega \mu _0 }, \\ \\ h_{\text {II}} = j \omega \mu _0. \end{matrix} \end{aligned}$$

Here, $k_x = \pi /a$ is the cutoff wavenumber in the *x* direction; *a* is the broader direction of the waveguide; $k_0$ is the free-space (unbounded) wavenumber; and *ω* is the angular frequency. In a similar fashion, $[\Gamma _{\text {III}}]$ for the substrate region only can be written as

4 $$\begin{aligned} {[}\Gamma _{\text {III}}] = \begin{bmatrix} 0 & 0 & 0 & 0 \\ 0 & 0 & h_{\text {III}} & 0 \\ 0 & g_{\text {III}} & 0 & 0 \\ 0 & 0 & 0 & 0 \end{bmatrix}, \ \ \ \ \begin{matrix} g_{\text {III}} = j \dfrac{ (k_0^2 \varepsilon _{rp} - k_x^2) }{ \omega \mu _0 }, \\ \\ h_{\text {III}} = j \omega \mu _0 = h_{\text {II}}. \end{matrix} \end{aligned}$$

The corresponding state-transition matrices for the sample and substrate regions ($[\Phi _{\text {II}}]$ and $[\Phi _{\text {III}}]$) can be derived as

5 $$\begin{aligned} {[}\Phi _{\text {II / III}}] = e^{[A_{(\text {II / III})}]} = \begin{bmatrix} 1 & 0 & 0 & 0 \\ 0 & \Phi _{22}^{(\text {II / III})} & \Phi _{23}^{(\text {II / III})} & 0 \\ 0 & \Phi _{32}^{(\text {II / III})} & \Phi _{22}^{(\text {II / III})} & 0 \\ 0 & 0 & 0 & 1 \end{bmatrix}, \end{aligned}$$

where

6 $$\begin{aligned} {[}A_{(\text {II})}]&= - [\Gamma _{\text {II}}] L_{\text {sam}}, \ \ \ [A_{(\text {III})}] = - [\Gamma _{\text {III}}] L_{\text {sub}}, \end{aligned}$$

7 $$\begin{aligned} \Phi _{22}^{\text {(II / III)}}&= \frac{1}{2} \left( e^{\lambda _1^{\text {(II / III)}} } + e^{\lambda _3^{\text {(II / III)}} } \right) , \end{aligned}$$

8 $$\begin{aligned} \Phi _{23}^{\text {(II / III)}}&= - \sqrt{ \frac{h_{\text {II / III}}}{g_{\text {II / III}}} } \frac{1}{2} \left( e^{\lambda _1^{\text {(II / III)}} } - e^{\lambda _3^{\text {(II / III)}} } \right) , \end{aligned}$$

9 $$\begin{aligned} \Phi _{32}^{\text {(II / III)}}&= - \sqrt{ \frac{g_{\text {II / III}}}{h_{\text {II / III}}} } \frac{1}{2} \left( e^{\lambda _1^{\text {(II / III)}}} - e^{\lambda _3^{\text {(II / III)}}} \right) . \end{aligned}$$

Here, $\lambda _1^{\text {(II / III)}}$ and $\lambda _3^{\text {(II / III)}}$ are the eigenvalues of the $[\Phi _{\text {II}}]$ and $[\Phi _{\text {III}}]$ with the values

10 $$\begin{aligned} \lambda _1^{\text {(II)}}&= \lambda _2^{\text {(II)}} = \sqrt{g_{\text {II}} h_{\text {II}}} L_{\text {sam}}, \ \ \ \lambda _3^{\text {(II)}} = \lambda _4^{\text {(II)}} = - \lambda _1^{\text {(II)}}, \end{aligned}$$

11 $$\begin{aligned} \lambda _1^{\text {(III)}}&= \lambda _2^{\text {(III)}} = \sqrt{g_{\text {III}} h_{\text {III}}} L_{\text {sub}}, \ \ \ \lambda _3^{\text {(III)}} = \lambda _4^{\text {(III)}} = - \lambda _1^{\text {(III)}}, \end{aligned}$$

Then, the state-transition matrix of the two-layer structure ($[\Phi _{\text {T}}]$) in Fig. 1 for a wave incidence from port-1 (Forward) and port-2 (Backward) can be determined from

12 $$\begin{aligned} {[}\Phi _{\text {T}}^{(\text {Forward})}] = [\Phi _{\text {II}}] \cdot [\Phi _{\text {III}}], \ \ [\Phi _{\text {T}}^{(\text {Backward})}] = [\Phi _{\text {III}}] \cdot [\Phi _{\text {II}}] \end{aligned}$$

Incorporating $[\Phi _{\text {II}}]$ and $[\Phi _{\text {III}}]$ in (5) into (12) results in

13 $$\begin{aligned} {[}\Phi _{\text {T}}^{\text {(Forward)}}] = \begin{bmatrix} 1 & 0 & 0 & 0 \\ 0 & \Phi _{22}^{(\text {T})} & \Phi _{23}^{(\text {T})} & 0 \\ 0 & \Phi _{32}^{(\text {T})} & \Phi _{33}^{(\text {T})} & 0 \\ 0 & 0 & 0 & 1 \end{bmatrix}, \end{aligned}$$

14 $$\begin{aligned} {[}\Phi _{\text {T}}^{\text {(Backward)}}] = \begin{bmatrix} 1 & 0 & 0 & 0 \\ 0 & \Phi _{33}^{(\text {T})} & \Phi _{23}^{(\text {T})} & 0 \\ 0 & \Phi _{32}^{(\text {T})} & \Phi _{22}^{(\text {T})} & 0 \\ 0 & 0 & 0 & 1 \end{bmatrix}, \end{aligned}$$

where

15 $$\begin{aligned} \Phi _{22}^{(\text {T})}&= \Phi _{22}^{(\text {II})} \Phi _{22}^{(\text {III})} + \Phi _{23}^{(\text {II})} \Phi _{32}^{(\text {III})}, \end{aligned}$$

16 $$\begin{aligned} \Phi _{23}^{(\text {T})}&= \Phi _{22}^{(\text {II})} \Phi _{23}^{(\text {III})} + \Phi _{23}^{(\text {II})} \Phi _{22}^{(\text {III})}, \end{aligned}$$

17 $$\begin{aligned} \Phi _{32}^{(\text {T})}&= \Phi _{32}^{(\text {II})} \Phi _{22}^{(\text {III})} + \Phi _{22}^{(\text {II})} \Phi _{32}^{(\text {III})}, \end{aligned}$$

18 $$\begin{aligned} \Phi _{33}^{(\text {T})}&= \Phi _{32}^{(\text {II})} \Phi _{23}^{(\text {III})} + \Phi _{22}^{(\text {II})} \Phi _{22}^{(\text {III})}. \end{aligned}$$

#### Relation between state matrices and S-parameters

For a wave with $\bar{E}_i (x,z) = \hat{a}_y \text {sin} (k_x x) e^{-j k_z z}$ incident from Port-1 to the two-layer structure, one can determine^41,43,45–48^

19 $$\begin{aligned}&\begin{bmatrix} 0 \\ \text {sin} (k_x x) \\ \end{bmatrix} + \begin{bmatrix} E_x^r(x,0) \\ E_y^r(x,0) \\ \end{bmatrix} = \Bigg ( \begin{bmatrix} 1 & 0 \\ 0 & \Phi _{22}^{\text {(T)}} \\ \end{bmatrix} \\ \nonumber&+ \begin{bmatrix} 0 & 0 \\ \Phi _{23}^{\text {(T)}} & 0\\ \end{bmatrix} \cdot [Z_0]^{-1} \Bigg ) \cdot \begin{bmatrix} E_x^t (x, L_{\text {sam}} + L_{\text {sub}}) \\ E_y^t (x, L_{\text {sam}} + L_{\text {sub}}) \\ \end{bmatrix}, \end{aligned}$$

20 $$\begin{aligned}&{[}Z_0]^{-1} \cdot \begin{bmatrix} 0 \\ \text {sin} (k_x x) \\ \end{bmatrix} - [Z_0]^{-1} \cdot \begin{bmatrix} E_x^r(x, 0) \\ E_y^r(x, 0) \\ \end{bmatrix} = \Bigg ( \begin{bmatrix} 0 & \Phi _{32}^{\text {(T)}} \\ 0 & 0 \\ \end{bmatrix} \\ \nonumber&+ \begin{bmatrix} \Phi _{33}^{\text {(T)}} & 0 \\ 0 & 1\\ \end{bmatrix} \cdot [Z_0]^{-1} \Bigg ) \cdot \begin{bmatrix} E_x^t (x, L_{\text {sam}} + L_{\text {sub}}) \\ E_y^t (x, L_{\text {sam}} + L_{\text {sub}}) \\ \end{bmatrix}, \end{aligned}$$

where *z= 0* is referenced to the front face of the sample and

21 $$\begin{aligned} {[}Z_0] = \begin{bmatrix} 0 & Z_{10} \\ -Z_{10} & 0\\ \end{bmatrix}, \ \ \ Z_{10} = \frac{\omega \mu _0}{\sqrt{k_0^2 - k_x^2}}. \end{aligned}$$

Using (19) and (20), one can derive

22 $$\begin{aligned} S_{11}^{\text {(T)}}&= \frac{ \Phi _{22}^{\text {(T)}} - \Phi _{33}^{\text {(T)}} + Z_{10} \Phi _{32}^{\text {(T)}} - \Phi _{23}^{\text {(T)}}/Z_{10} }{ \Phi _{22}^{\text {(T)}} + \Phi _{33}^{\text {(T)}} - Z_{10} \Phi _{32}^{\text {(T)}} - \Phi _{23}^{\text {(T)}}/Z_{10} }, \end{aligned}$$

23 $$\begin{aligned} S_{21}^{\text {(T)}}&= \frac{ 2 }{ \Phi _{22}^{\text {(T)}} + \Phi _{33}^{\text {(T)}} - Z_{10} \Phi _{32}^{\text {(T)}} - \Phi _{23}^{\text {(T)}}/Z_{10} }. \end{aligned}$$

Following the above procedure and assuming a wave with $\bar{E}_i (x,z) = \hat{a}_y \text {sin} (k_x x) e^{+j k_z z}$ incident from Port-2 to the two-layer structure, one can find

24 $$\begin{aligned} S_{22}^{\text {(T)}}&= \frac{ -\Phi _{22}^{\text {(T)}} + \Phi _{33}^{\text {(T)}} + Z_{10} \Phi _{32}^{\text {(T)}} - \Phi _{23}^{\text {(T)}}/Z_{10} }{ \Phi _{22}^{\text {(T)}} + \Phi _{33}^{\text {(T)}} - Z_{10} \Phi _{32}^{\text {(T)}} - \Phi _{23}^{\text {(T)}}/Z_{10} }, \end{aligned}$$

25 $$\begin{aligned} S_{12}^{\text {(T)}}&= \frac{ 2 }{ \Phi _{22}^{\text {(T)}} + \Phi _{33}^{\text {(T)}} - Z_{10} \Phi _{32}^{\text {(T)}} - \Phi _{23}^{\text {(T)}}/Z_{10} } = S_{21}^{\text {(T)}}. \end{aligned}$$

### Extraction procedure

The proposed extraction procedure for determination of $\varepsilon _{rs}$ relies on the following two steps. The first step involves evaluation of $\Phi _{22}^{\text {(T)}}$, $\Phi _{23}^{\text {(T)}}$, $\Phi _{32}^{\text {(T)}}$, and $\Phi _{33}^{\text {(T)}}$ from measured $S_{11}^{\text {(T)}}$, $S_{21}^{\text {(T)}} = S_{12}^{\text {(T)}}$, and $S_{22}^{\text {(T)}}$ of the two-layer structure in Fig. 1. From (22), (23), and (25), one determines $\Phi _{23}^{\text {(T)}}$, $\Phi _{32}^{\text {(T)}}$, and $\Phi _{33}^{\text {(T)}}$ in terms of $\Phi _{22}^{\text {(T)}}$

26 $$\begin{aligned} \Phi _{23}^{\text {(T)}}&= Z_{10} \left( \Phi _{22}^{\text {(T)}} -\frac{ \left( S_{11}^{\text {(T)}} + 1 \right) }{S_{21}^{\text {(T)}}} \right) , \end{aligned}$$

27 $$\begin{aligned} \Phi _{32}^{\text {(T)}}&= \frac{1}{Z_{10}} \left( \Phi _{22}^{\text {(T)}} + \frac{ \left( S_{22}^{\text {(T)}} - 1 \right) }{S_{21}^{\text {(T)}}} \right) , \end{aligned}$$

28 $$\begin{aligned} \Phi _{33}^{\text {(T)}}&= \Phi _{22}^{\text {(T)}} + \frac{ \left( S_{22}^{\text {(T)}} - S_{11}^{\text {(T)}} \right) }{S_{21}^{\text {(T)}}}. \end{aligned}$$

This means that an additional expression is needed to determine $\Phi _{22}^{\text {(T)}}$ from which $\Phi _{23}^{\text {(T)}}$, $\Phi _{32}^{\text {(T)}}$, and $\Phi _{33}^{\text {(T)}}$ can be found.

It is shown in^41–47^ that the determinant $\text {det} (\cdot )$ of $[\Phi _{\text {T}}^{\text {(Forward)}}]$ and $[\Phi _{\text {T}}^{\text {(Backward)}}]$ can be written as

29 $$\begin{aligned} \text {det} \left( [\Phi _{\text {T}}^{\text {(Forward)}}] \right)&= \text {det} \left( [\Phi _{\text {II}}] \right) \text {det} \left( [\Phi _{\text {III}}] \right) = 1, \end{aligned}$$

30 $$\begin{aligned} \text {det} \left( [\Phi _{\text {T}}^{\text {(Backward)}}] \right)&= \text {det} \left( [\Phi _{\text {III}}] \right) \text {det} \left( [\Phi _{\text {II}}] \right) = 1, \end{aligned}$$

because

31 $$\begin{aligned}&\text {det} \left( [\Phi _{\text {II}}] \right) = \text {det} \left( [V_{\text {II}}] \right) \text {det} \left( e^{[A_{\text {II}}]} \right) \text {det} \left( [V_{\text {II}}]^{-1} \right) \nonumber \\&= e^{\left[ \lambda _1^{\text {(II)}} + \lambda _2^{\text {(II)}} + \lambda _3^{\text {(II)}} + \lambda _4^{\text {(II)}} \right] L_{\text {sam}} } = 1, \end{aligned}$$

32 $$\begin{aligned}&\text {det} \left( [\Phi _{\text {III}}] \right) = \text {det} \left( [V_{\text {III}}] \right) \text {det} \left( e^{[A_{\text {III}}]} \right) \text {det} \left( [V_{\text {III}}]^{-1} \right) \nonumber \\&= e^{\left[ \lambda _1^{\text {(III)}} + \lambda _2^{\text {(III)}} + \lambda _3^{\text {(III)}} + \lambda _4^{\text {(III)}} \right] L_{\text {sub}} } = 1, \end{aligned}$$

From (13)-(18), it can be readily determined that

33 $$\begin{aligned} \Phi _{22}^{\text {(T)}} \Phi _{33}^{\text {(T)}} - \Phi _{23}^{\text {(T)}} \Phi _{32}^{\text {(T)}} = 1. \end{aligned}$$

Substituting (26)-(28) results in

34 $$\begin{aligned} \Phi _{22}^{\text {(T)}} = \frac{ S_{21}^{\text {(T)}} S_{12}^{\text {(T)}} + \left( 1 + S_{11}^{\text {(T)}} \right) \left( 1 - S_{22}^{\text {(T)}} \right) }{ 2 S_{21}^{\text {(T)}} }. \end{aligned}$$

It is then feasible to compute $\Phi _{23}^{\text {(T)}}$, $\Phi _{32}^{\text {(T)}}$, and $\Phi _{33}^{\text {(T)}}$ from (26)-(28). The advantage of our analysis over those in the studies^41–45^ is that we have derived closed-form expressions between S-parameters and state vector.

Then, the second step entails extraction of $\varepsilon _{rs}$ from (15)-(18) along with (7)-(9). After incorporating (7)-(9) into (15)-(18), one can obtain

35 $$\begin{aligned} \Phi _{22}^{(\text {T})}&= \Omega _1 + \sqrt{ h_{\text {II}}/g_{\text {II}} } \sqrt{ g_{\text {III}}/h_{\text {III}} } \Omega _2, \end{aligned}$$

36 $$\begin{aligned} \Phi _{23}^{(\text {T})}&= - \sqrt{ h_{\text {III}} / g_{\text {III}} } \Omega _3 - \sqrt{ h_{\text {II}} / g_{\text {II}} } \Omega _4, \end{aligned}$$

37 $$\begin{aligned} \Phi _{32}^{(\text {T})}&= - \sqrt{ g_{\text {III}} / h_{\text {III}} } \Omega _3 - \sqrt{ g_{\text {II}} / h_{\text {II}} } \Omega _4, \end{aligned}$$

38 $$\begin{aligned} \Phi _{33}^{(\text {T})}&= \Omega _1 + \sqrt{ g_{\text {II}}/h_{\text {II}} } \sqrt{ h_{\text {III}}/g_{\text {III}} } \Omega _2, \end{aligned}$$

where

39 $$\begin{aligned} \Omega _1&= \text {cosh} (\lambda _1^{\text {(II)}}) \text {cosh} (\lambda _1^{\text {(III)}}), \Omega _2 = \text {sinh} (\lambda _1^{\text {(II)}}) \text {sinh} (\lambda _1^{\text {(III)}}) \end{aligned}$$

40 $$\begin{aligned} \Omega _3&= \text {cosh} (\lambda _1^{\text {(II)}}) \text {sinh} (\lambda _1^{\text {(III)}}), \Omega _4 = \text {sinh} (\lambda _1^{\text {(II)}}) \text {cosh} (\lambda _1^{\text {(III)}}). \end{aligned}$$

**Fig. 2:**
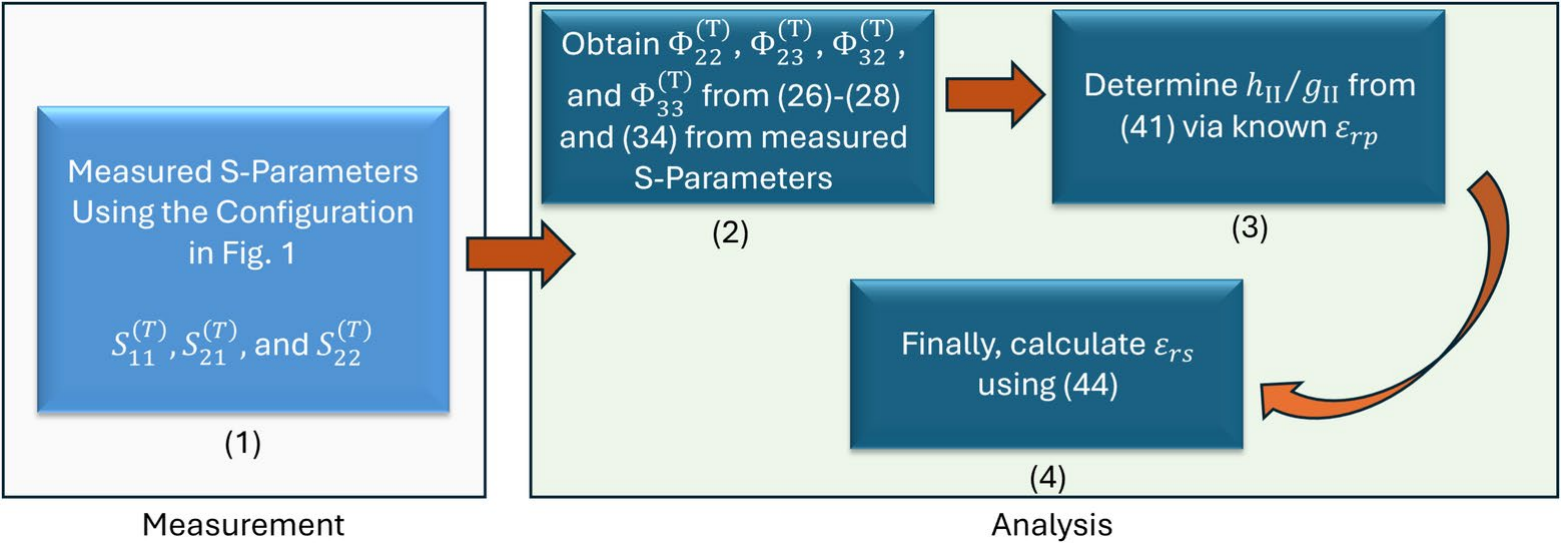
Flowchart of the proposed method.

After some manipulations, the following expression can be derived from (35) and (38)

41 $$\begin{aligned} \frac{h_{\text {II}}}{g_{\text {II}}} = \frac{ \Phi _{22}^{\text {(T)}} \frac{h_{\text {III}}}{g_{\text {III}}} \text {sinh} (\lambda _1^{\text {(III)}}) + \text {cosh} (\lambda _1^{\text {(III)}}) \Phi _{23}^{\text {(T)}} \sqrt{ \frac{h_{\text {II}}}{g_{\text {II}}} } }{ \Phi _{33}^{\text {(T)}} \text {sinh} (\lambda _1^{\text {(III)}}) + \text {cosh} (\lambda _1^{\text {(III)}}) \Phi _{32}^{\text {(T)}} \sqrt{ \frac{h_{\text {II}}}{g_{\text {II}}} } }. \end{aligned}$$

It is seen from (41) that $h_{\text {II}}/g_{\text {II}}$ requires $L_{\text {sub}}$ information. Then, from (36) and (37), one can obtain

42 $$\begin{aligned} \frac{\text {sinh} (\lambda _1^{\text {(III)}})}{\text {cosh} (\lambda _1^{\text {(III)}})} = \frac{ \left( \Phi _{22}^{\text {(T)}} - \Phi _{33}^{\text {(T)}} \right) \sqrt{ \frac{h_{\text {II}}}{g_{\text {II}}} } }{ \frac{h_{\text {II}}}{g_{\text {II}}} \Phi _{32}^{\text {(T)}} - \Phi _{23}^{\text {(T)}}}. \end{aligned}$$

Substituting (42) back into (41) yields

43 $$\begin{aligned} \frac{h_{\text {II}}}{g_{\text {II}}} = \frac{ \left[ \Phi _{22}^{\text {(T)}} \left( \Phi _{22}^{\text {(T)}} - \Phi _{33}^{\text {(T)}} \right) + \Phi _{23}^{\text {(T)}} \Phi _{32}^{\text {(T)}} \right] \frac{h_{\text {III}}}{g_{\text {III}}} - {\Phi _{23}^{\text {(T)}} }^2 }{ {\Phi _{32}^{\text {(T)}} }^2 \frac{h_{\text {III}}}{g_{\text {III}}} + \left[ \Phi _{33}^{\text {(T)}} \left( \Phi _{22}^{\text {(T)}} - \Phi _{33}^{\text {(T)}} \right) - \Phi _{23}^{\text {(T)}} \Phi _{32}^{\text {(T)}} \right] }. \end{aligned}$$

Finally, $\varepsilon _{rs}$ can be calculated from

44 $$\begin{aligned} \varepsilon _{rs} = \eta _0^2/(h_{\text {II}}/g_{\text {II}}) + \left( f_c/f \right) ^2 \end{aligned}$$

where $f_c$ and *f* are the cutoff and operating frequencies and

45 $$\begin{aligned} h_{\text {III}}/g_{\text {III}} = \eta _0^2/[ \varepsilon _{rp} - \left( f_c/f \right) ^2 ]. \end{aligned}$$

Flow chart for the implementation of the proposed method is presented in Fig. 2.

It is noteworthy that our proposed extraction method relies on $\varepsilon _{rp}$ information only, unlike the extraction methods in the studies^27–35^. Besides, different from the method in the study^36^, our extraction method does not necessitate the application of the time-gating procedure, which inherently introduces undesired ripples at the initial and final frequencies in the band. Furthermore, our derivation here considers a two-layer configuration in Fig. 1, different than the one-layer configuration in the studies^46,47^.

**Fig. 3:**
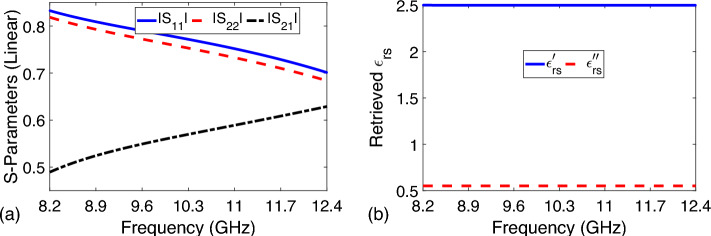
(**a**) S-Parameters and (**b**) retrieved $\varepsilon _{rs}$ of the considered two-layer structure (Ideal Case).

### Validation and accuracy analysis

Numerical analysis was performed to validate our proposed method. For this end, the following parameters were assumed: $\varepsilon _{rs} = 2.50 - j 0.55$, $\varepsilon _{rp} = 6.0 - j 0.45$, $L_{\text {sam}} = 1.00$ mm, and $L_{\text {sub}} = 2.84$ mm (X-band with $f_c = 6.557$ GHz). These parameters were selected considering the tested materials in Sect. 3. Figure 3a and b illustrate, respectively, the calculated $|S_{11}^{\text {(T)}}|$, $|S_{21}^{\text {(T)}}|$, and $|S_{22}^{\text {(T)}}|$ over frequency and retrieved $\varepsilon _{rs}$ using our proposed method. It is noted from Fig. 3a that, as expected, $|S_{11}^{\text {(T)}}|$ and $|S_{22}^{\text {(T)}}|$ are different for the asymmetric two-layer structure in Fig. 1. Additionally, retrieved $\varepsilon _{rs}$ by the proposed method is good agreement with that synthesized of the considered two-layer structure over the entire frequency band, validating our proposed method.

**Fig. 4:**
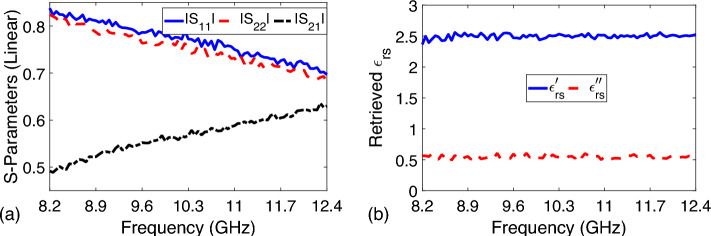
(**a**) S-Parameters and (**b**) retrieved $\varepsilon _{rs}$ of the considered two-layer structure after some white noise with an SNR value of 25 dB was added to all S-parameters.

The previous numerical analysis can be considered as an ideal case because calculated S-parameters were noise-free. To imitate a real scenario, white Gaussian noise was added to calculated S-parameters. Figure 4a and b demonstrate, respectively, the calculated $|S_{11}^{\text {(T)}}|$, $|S_{21}^{\text {(T)}}|$, and $|S_{22}^{\text {(T)}}|$ over frequency and retrieved $\varepsilon _{rs}$ using our proposed method when some white noise with an SNR value of 25 dB was superimposed on all calculated S-parameters. It is noted that this level of SNR value is comparatively low for modern VNA instruments. In implementation this noise, ‘awgn’ functions of MATLAB© was utilized. This added noise introduces ripples in the calculated $|S_{11}^{\text {(T)}}|$, $|S_{21}^{\text {(T)}}|$, and $|S_{22}^{\text {(T)}}|$ as seen from Fig. 4a. It is also noted from Fig. 4b that retrieved $\varepsilon _{rs}$ by the proposed method partially oscillates around (with a maximum difference less than 5%) but still follows the actual value of $\varepsilon _{rs} = 2.50 - j 0.55$.

The final analysis takes into account of the effect of any air gap with length $L_{\text {gap}}$ present between the sample and the substrate on extracted $\varepsilon _{rs}$ by the proposed method. For this analysis, $[\Phi _{\text {III}}]$ was replaced with

46 $$\begin{aligned} {[}\Phi _{\text {III}}] = [\Phi _0] \cdot [\Phi _{\text {III}}] \end{aligned}$$

where

47 $$\begin{aligned} {[}\Phi _0]&= e^{- [\Gamma _0] L_{\text {gap}}} = \begin{bmatrix} 1 & 0 & 0 & 0 \\ 0 & \Phi _{22}^{(0)} & \Phi _{23}^{(0)} & 0 \\ 0 & \Phi _{32}^{(0)} & \Phi _{22}^{(0)} & 0 \\ 0 & 0 & 0 & 1 \end{bmatrix}, \end{aligned}$$

48 $$\begin{aligned} {[}\Gamma _0]&= \begin{bmatrix} 0 & 0 & 0 & 0 \\ 0 & 0 & h_0 & 0 \\ 0 & g_0 & 0 & 0 \\ 0 & 0 & 0 & 0 \end{bmatrix}, \ \ \ \ \begin{matrix} g_0 = j \dfrac{ (k_0^2 - k_x^2) }{ \omega \mu _0 }, \\ \\ h_0 = j \omega \mu _0. \end{matrix} \end{aligned}$$

**Fig. 5:**
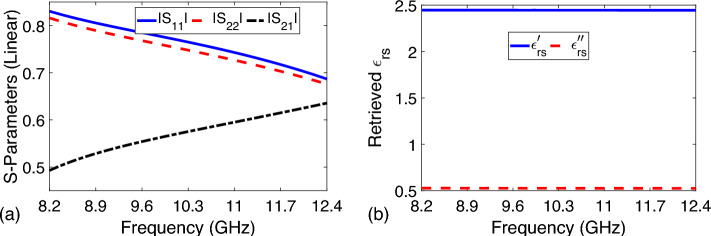
(**a**) S-Parameters and (**b**) retrieved $\varepsilon _{rs}$ of the considered two-layer structure when air gap ($L_{\text {gap}} = 0.2$ mm) between the sample and the substrate is present.

**Fig. 6:**
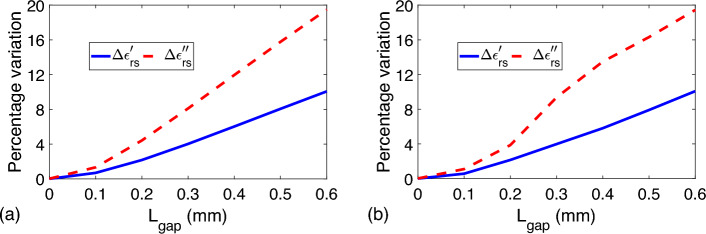
Percentage variation of extracted $\varepsilon _{rs}$ (averaged over the entire frequency) versus $L_{\text {gap}}$ (**a**) when there is no noise effect (SNR$\rightarrow \infty$) and (**b**) when white noise with an SNR value of 25 dB was added to all S-parameters.

In the analysis of the effect of any potential air gap on $\varepsilon _{rs}$ extraction, $[\Phi _{\text {T}}^{\text {Forward}}]$ and $[\Phi _{\text {T}}^{\text {Backward}}]$ were first determined from (12) using (46). Then, our extraction method is implemented to determine $\varepsilon _{rs}$ with new S-parameters involving the effect of air gap. Figure 5a and b present, respectively, the calculated $|S_{11}^{\text {(T)}}|$, $|S_{21}^{\text {(T)}}|$, and $|S_{22}^{\text {(T)}}|$ over frequency and retrieved $\varepsilon _{rs}$ using our proposed method when $L_{\text {gap}} = 0.2$ mm, which is 20% of the sample length. While this air gap results in a partial reduction in $|S_{11}^{\text {(T)}}|$ and $|S_{22}^{\text {(T)}}|$, it produces a slight increase in $|S_{21}^{\text {(T)}}|$. This is because presence of air gap reduces the effective impedance of either the sample or the substrate (lower reflection) and increases transmission according to the power conservation principle (air is a lossless medium). Besides, it is seen from Fig. 5b that extracted real and imaginary parts of $\varepsilon _{rs}$ are lower since air gap reduces the effective dielectric constant and loss factor of the sample (sample-air combination). For a quantitative comparison, percentage variations of real and imaginary parts of extracted $\varepsilon _{rs}$ (averaged by the entire frequency band) versus $L_{\text {gap}}$ are drawn in Fig. 6a. It is seen from Fig. 6a that $\Delta \varepsilon _{rs}^{\prime }$ and $\Delta \varepsilon _{rs}^{\prime \prime }$ increase with $L_{\text {gap}}$ mainly due to increased variation in $|S_{11}^{\text {(T)}}|$, $|S_{21}^{\text {(T)}}|$, and $|S_{22}^{\text {(T)}}|$ from their nominal values (when $L_{\text {gap}} = 0.0$). Finally, $\Delta \varepsilon _{rs}^{\prime }$ and $\Delta \varepsilon _{rs}^{\prime \prime }$ versus $L_{\text {gap}}$ were determined when some white noise with an SNR value of 25 dB was superimposed on all calculated S-parameters. It is observed from Fig. 6a and b that added noise barely changes $\Delta \varepsilon _{rs}^{\prime }$ and $\Delta \varepsilon _{rs}^{\prime \prime }$ over the entire $L_{\text {gap}}$ values, manifesting that our proposed method is comparatively resistant to white Gaussian noise if average $\varepsilon _{rs}$ is concerned.

## Results and discussions

### Microwave experimental setup

**Fig. 7:**
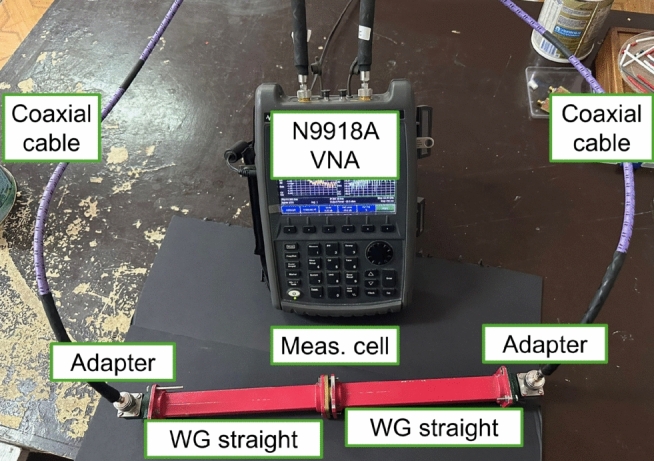
A photo showing the measurement setup used for validation.

Fig. 7 illustrates the waveguide (X-band - 8.2:12.4 GHz with a cross section of *22.86 × 10.16* mm$^2$) measurement setup used to validate our proposed method. It has a vector network analyzer (VNA) from Keysight Technologies (Model: N9918A), two waveguide adapters from Flann Microwave Instruments, two standard waveguide straights, one measurement cell with a length of 9.40 mm, and two 3.5 mm rugged phase stable coaxial cables with a length of 100 cm. The VNA has a frequency band of operation from near DC (30 kHz) up to 26.5 GHz with a higher dynamic range of approximately 75 dB. It is in charge of generating and measuring two-port S-parameters. The waveguide straights between the adapters and the cell are utilized to mitigate the effect of higher order modes, if any, in the measurement system. The overall measurement system was calibrated by the Thru-Reflect-Line (TRL) calibration technique^49^ because implementation of these standards at microwave frequencies are relatively easier. While the measurement cell (a length of 9.40 mm) was utilized as the line standard, a highly-reflective (its specific value is not needed) metal termination was operated as the reflect standard. Thru standard was implementing by connecting the waveguide straights together (zero length line). Before actual measurements, preliminary measurements were carried out to validate the implemented TRL calibration procedure using these standards. It was recorded that magnitudes of reflection S-parameters of the thru or line standard were lower than *-40* dB over the entire frequency band, and magnitudes of transmission S-parameters of the thru or line standard were bigger than *-0.01* dB over the entire frequency band. Furthermore, it was noted that phases of the transmission S-parameters of the thru standard were nearly zero over the entire frequency band while phases of the transmission S-parameters of the line standard were around $-90^o$ at the mid frequency (10.3 GHz). These metrics are considered to be sufficient requirements to validate the accuracy of the TRL procedure^49^. Measurements with 1001 frequency points over X-band were carried out at ordinary laboratory conditions (temperature: *23 ∓ 1* $^o$C and relative humidity: *50 - 60*%). Detailed information of the measurement setup can be found from the studies^30,34,35^.

### About implemented extraction methods, selection of substrate properties, and sample/Substrate positioning

Different one-port and two-port methods were applied to assess the performance of the proposed method for various cases considered below. While the methods in the studies^23,29,30,32,35^ rely on two-port reflection and transmission S-parameter measurements, the method in the study^34^ utilizes one-port reflection-only S-parameter measurements. These methods were selected considering whether they are applicable for $\varepsilon _{rs}$ determination of a two-layer configuration and having a repertoire of methods with different limitations (whether they require sample thickness and/or substrate thickness). It is noteworthy that the methods in the studies^1,36^ were not applicable for X-band waveguide measurements, because the bandwidth is not sufficiently broader for the implementation of time-gating procedure to isolate relevant first reflection/transmission terms needed for accurate $\varepsilon _{rs}$ retrieval for these methods. For each method, measurements were repeated five times for each measurement case considered below to monitor their repeatability. Quantitative analysis of these results are presented in Subsect. 3.7.

On the other hand, as depicted in Fig. 2, it is instructive to note that our proposed extraction method utilizes $\varepsilon _{rp}$ and $L_{\text {sub}}$ as input parameters to evaluate $\varepsilon _{rs}$. Therefore, any inaccuracy in $\varepsilon _{rp}$ and $L_{\text {sub}}$ input parameters can influence such an evaluation. In our recent study^34^, expressions for examining dependencies of $\varepsilon _{rs}$ versus and $\varepsilon _{rp}$ and $L_{\text {sub}}$ for sensitivity analysis have been derived. It is noteworthy that although such sensitivity analysis is a function of $\varepsilon _{rs}$, $\varepsilon _{rp}$, $L_{\text {sam}}$, $L_{\text {sub}}$, and frequency (or frequency band) in complex manner, the following remarks can be considered as a rule of thumb. First, $L_{\text {sub}}$ should be relatively thicker so that sample will not have sagging. Similarly, it should not be large to allow sufficient wave propagation through the sample. Second, $\varepsilon _{rp}$ should have moderate (small) loss if the sample under examination is low-loss (high-loss) so that electromagnetic signals will produce different $S_{11}^{\text {(T)}}$ and $S_{22}^{\text {(T)}}$ reactions, the level of $|S_{21}^{\text {(T)}}| \ge -40$ dB (above the noise level of most modern microwave VNA instruments), and numerical stability will be improved.

Finally, it is noted that small air gaps are needed between the waveguide cross section and sample/substrate transverse surfaces for a flexible positioning of sample/substrate structure. Recent studies^50,51^ have demonstrated that air gaps needed for convenient positioning could be tailored at the left and right sides of the sample/substrate without much altering pattern of electric and magnetic fields, because electric fields mainly concentrate around center of the guide in the broader dimension. The samples/substrates tested in our proposed method were machined in such manner that they have a transverse dimension of around *22.45 .14* mm$^2$.

### Case-I:results for known sample thickness and substrate thickness

For this measurement, a PVC sample with $L_{\text {sam}} \cong 1.00$ mm was positioned over a glass substrate material (soda-lime glass) with $L_{\text {sub}} \cong 2.84 \mp 0.083$ mm measured by a high precision caliper (Mitutoyo with a dial reading) with a resolution of 0.05 mm over 200 mm scale. Its permittivity $\varepsilon _{rp}$ was obtained as *≅ 6.00 - j 0.05* (with an uncertainty less than approximately 3% over for both of its real and imaginary parts over the entire frequency band) by using the method^24^. It is assumed that measured S-parameters were referenced to the sample left surface and substrate right surface. After considering the effect of this gap analyzed in Sect. 2.3 (Fig. 6a and b), a special care was exercised in eliminating any possible air gap between the sample and its substrate while performing $\varepsilon _{rs}$ measurements of this PVC sample for the examined case and other cases that will be discussed shortly.

**Fig. 8:**
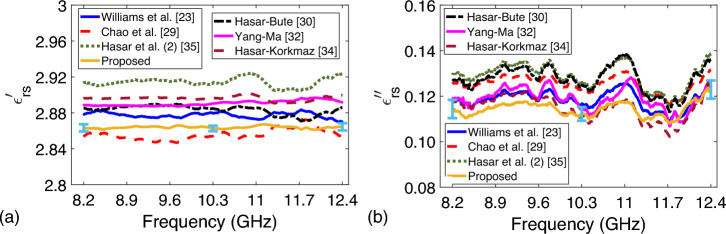
Extracted (**a**) $\varepsilon _{rs}^{\prime }$ and (**b**) $\varepsilon _{rs}^{\prime \prime }$ of the PVC sample with $L_{\text {sam}} = 1.00$ mm for the Case-I (Ideal) by the Williams et al. method in the study^23^, the Chao et al. method in the study^29^, the Hasar et al. (2) method in the study^35^, the Hasar-Bute method in the study^30^, the Yang-Ma method in the study^32^, the Hasar-Korkmaz method in the study^34^, and the proposed method [Error bars for the extracted parameters by the proposed method are given at discrete frequencies 8.2 GHz (minimum frequency), 10.3 GHz (mid-frequency), and 12.4 GHz (maximum frequency)].

Our proposed method, the Williams et al. method in the study^23^, the Chao et al. method in the study^29^, the Hasar et al. (2) method in the study^35^, the Hasar-Bute method in the study^30^, the Yang-Ma method in the study^32^, and the Hasar-Korkmaz method in the study^34^ were applied for determining the real and imaginary parts of $\varepsilon _{rs}$ of PVC sample, as shown in Fig. 8a and b. It is observed from the results in Fig. 8a and b that extracted $\varepsilon _{rs}^{\prime }$ and $\varepsilon _{rs}^{\prime \prime }$ of the PVC sample by the Williams et al. method in the study^23^, the Chao et al. method in the study^29^, the Hasar et al. (2) method in the study^35^, the Hasar-Bute method in the study^30^, the Yang-Ma method in the study^32^, the Hasar-Korkmaz method in the study^34^, and the proposed method are in good agreement with each other over the entire frequency band and are in line with the reference datum of $\varepsilon _{rs} \cong 2.705 - j0.007$ for the PVC sample^52^ at room temperature and at X-band. Besides, extracted $\varepsilon _{rs}^{\prime \prime }$ of the PVC sample by all implemented methods are all greater than zero, which is inline with the passivity principle.

### Case-II:results for known sample thickness (unknown substrate thickness)

**Fig. 9:**
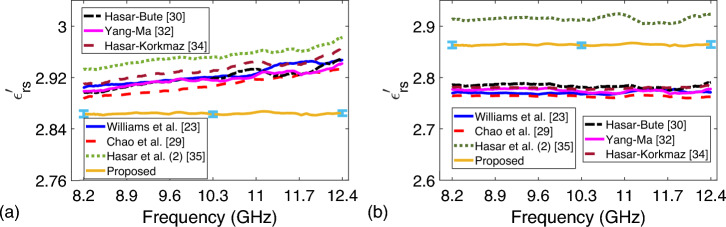
Extracted $\varepsilon _{rs}^{\prime }$ of the PVC sample with $L_{\text {sam}} = 1.00$ mm for (**a**) the Case-II ($L_{\text {sub}}$ was *-2*% offset) and (**b**) the Case-III ($L_{\text {sam}}$ was *+5*% offset) by the Williams et al. method in the study^23^, the Chao et al. method in the study^29^, the Hasar et al. (2) method in the study^35^, the Hasar-Bute method in the study^30^, the Yang-Ma method in the study^32^, the Hasar-Korkmaz method in the study^34^, and the proposed method [Error bars for the extracted parameters by the proposed method are given at discrete frequencies 8.2 GHz (minimum frequency), 10.3 GHz (mid-frequency), and 12.4 GHz (maximum frequency)].

This case was considered to examine the influence of unknown $L_{\text {sub}}$ on $\varepsilon _{rs}$ extraction. Fig. 9a demonstrates the extracted $\varepsilon _{rs}^{\prime }$ of the tested PVC sample using different methods. In implementation of each method, a *-2.0*% offset in $L_{\text {sub}}$ from its nominal/actual value ($L_{\text {sub}} = 2.84$ mm) was deliberately executed for such an analysis. Extracted $\varepsilon _{rs}^{\prime \prime }$ results of the tested PVC sample for this case are not presented for brevity. Quantitative analysis for both $\varepsilon _{rs}^{\prime }$ and $\varepsilon _{rs}^{\prime \prime }$ for this case will be presented later. It is seen from the results in Figs. 8a and 9a that while the Williams et al. method in the study^23^, the Chao et al. method in the study^29^, the Hasar et al. (2) method in the study^35^, the Hasar-Bute method in the study^30^, the Yang-Ma method in the study^32^, the Hasar-Korkmaz method in the study^34^ are all affected by imprecise $L_{\text {sub}}$ information, our proposed is not influenced. This is because while our proposed method does not require $L_{\text {sub}}$ information, the other tested methods are highly dependent on this information. For example, extracted $\varepsilon _{rs}^{\prime }$ at 10.3 GHz by the Williams et al. method in the study^23^ changes from around 2.88 to nearly 2.92 with a *+1.5*% percentage change for a *-2.0*% offset in $L_{\text {sub}}$. This clearly indicates the importance of the requirement of accurate $L_{\text {sub}}$ information for accurate $\varepsilon _{rs}$ extraction.

### Case-III:results for known substrate thickness (unknown sample thickness)

As a third case, the effect of unknown $L_{\text {sam}}$ on $\varepsilon _{rs}$ extraction was examined. Similar to the Case-II, *+5*% offset was intentionally applied on $L_{\text {sam}}$ from its nominal/actual value ($L_{\text {sam}} \cong 1.0$ mm). Figure 9b shows retrieved $\varepsilon _{rs}^{\prime }$ of the PVC sample by the Williams et al. method in the study^23^, the Chao et al. method in the study^29^, the Hasar et al. (2) method in the study^35^, the Hasar-Bute method in the study^30^, the Yang-Ma method in the study^32^, the Hasar-Korkmaz method in the study^34^, and the proposed method. Extracted $\varepsilon _{rs}^{\prime \prime }$ results of the tested PVC sample for this case are not presented for conciseness. Quantitative analysis for both $\varepsilon _{rs}^{\prime }$ and $\varepsilon _{rs}^{\prime \prime }$ for this case will be presented later. It is noticed from the results in Figs. 8a and 9b that extracted $\varepsilon _{rs}^{\prime }$ of the PVC sample by the Williams et al. method in the study^23^, the Chao et al. method in the study^29^, the Hasar-Bute method in the study^30^, the Yang-Ma method in the study^32^, the Hasar-Korkmaz method in the study^34^ are seriously affected by this offset. For example, a +5% offset in $L_{\text {sam}}$ introduces a change in $\varepsilon _{rs}^{\prime }$ from 2.854 to 2.762 for PVC sample (approximately *8.1%* change) for the Chao et al. method in the study^29^. On the other hand, determined $\varepsilon _{rs}^{\prime }$ of the PVC sample by the Hasar et al. (2) method in the study^35^ and the proposed method are not influenced because they do not necessitate $L_{\text {sam}}$ for $\varepsilon _{rs}$ extraction. Similar to the Case-II, any method not dependent on $L_{\text {sam}}$ is a necessity when its precise value is not known or when thickness uncertainty plays a key role for thinner samples.

### Case IV:effects of reference-plane information

**Fig. 10:**
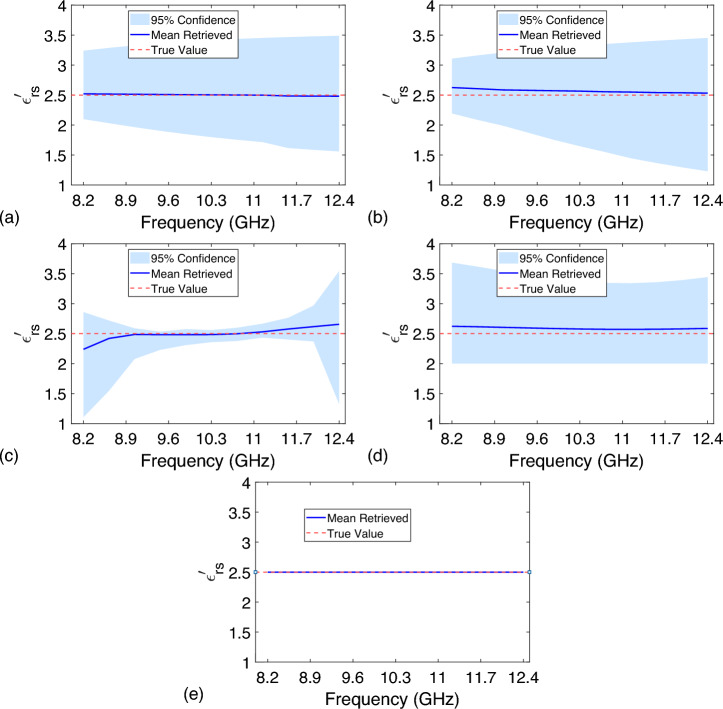
Extracted $\varepsilon _{rs}^{\prime }$ of the considered two-layer structure by (**a**) the Yang-Ma method in the study^32^, (**b**) the Hasar-1 method in the study^27^, (**c**) the Hasar et al. (2) method in the study^35^, (**d**) the Williams et al. method in the study^23^, and (**e**) the proposed method when *+5%* offset was introduced to the substrate thickness ($L_{\text {sub}}$).

Although our proposed method is an effective tool for evaluating $\varepsilon _{rs}$ of thinners dielectric samples positioned on a substrate material when $L_{\text {sub}}$ and $L_{\text {sam}}$ are not known precisely, it necessitates precise location of the sample on the substrate within a measurement cell (reference-plane information). This need arises whenever the overall sample and substrate thickness (arbitrarily positioned) is less than the length of the measurement cell. For the former three cases, the sample was flushed with the left termination of the measurement cell (9.40 mm) so that no reference plane information was needed. To evaluate the performance of the proposed method when imprecise reference plane information is present, a small offset (1.00 mm) between the sample and the left termination of the measurement cell was introduced. Figure 10a presents extracted $\varepsilon _{rs}^{\prime }$ of the PVC sample by the Williams et al. method in the study^23^, the Chao et al. method in the study^29^, the Hasar et al. (2) method in the study^35^, the Hasar-Bute method in the study^30^, the Yang-Ma method in the study^32^, the Hasar-Korkmaz method in the study^34^, and the proposed method. Extracted $\varepsilon _{rs}^{\prime \prime }$ results of the tested PVC sample for this case are not presented for conciseness. Quantitative analysis for both $\varepsilon _{rs}^{\prime }$ and $\varepsilon _{rs}^{\prime \prime }$ for this case will be discussed shortly. It is seen from the results in Figs. 8a and 10a that while extracted $\varepsilon _{rs}^{\prime }$ of the PVC sample by the Hasar et al. (2) method in the study^35^, the Hasar-Bute method in the study^30^, and the Hasar-Korkmaz method in the study^34^ are not influenced with this offset, those extracted by the Williams et al. method in the study^23^, the Chao et al. method in the study^29^, the Yang-Ma method in the study^32^, and the proposed method are altered by this offset. This is because the Williams et al. method in the study^23^, the Chao et al. method in the study^29^, the Yang-Ma method in the study^32^, and the proposed method are dependent on the information about the precise location of the sample on the substrate within the measurement cell.

**Fig. 11:**
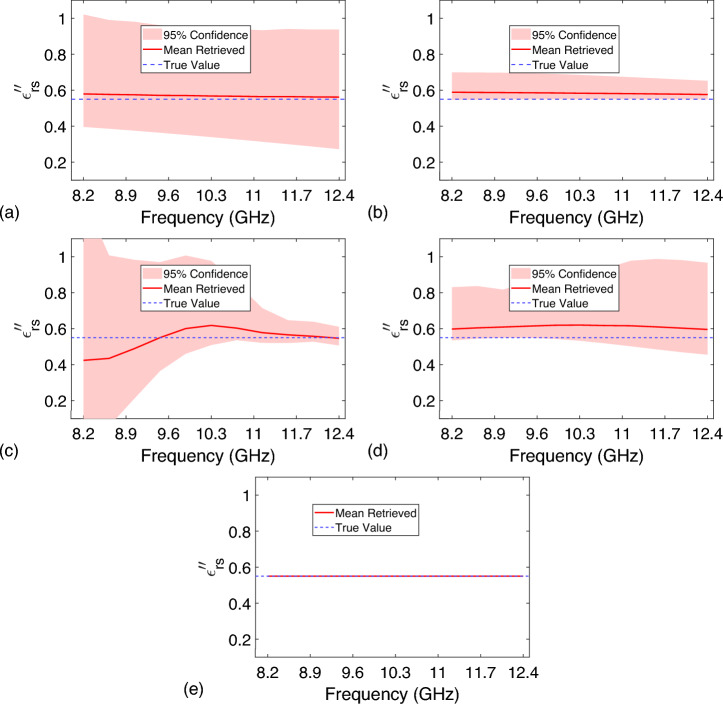
Extracted $\varepsilon _{rs}^{\prime \prime }$ of the considered two-layer structure by (**a**) the Yang-Ma method in the study^32^, (**b**) the Hasar-1 method in the study^27^, (**c**) the Hasar et al. (2) method in the study^35^, (**d**) the Williams et al. method in the study^23^, and (**e**) the proposed method when *+5%* offset was introduced to the substrate thickness ($L_{\text {sub}}$).

**Fig. 12:**
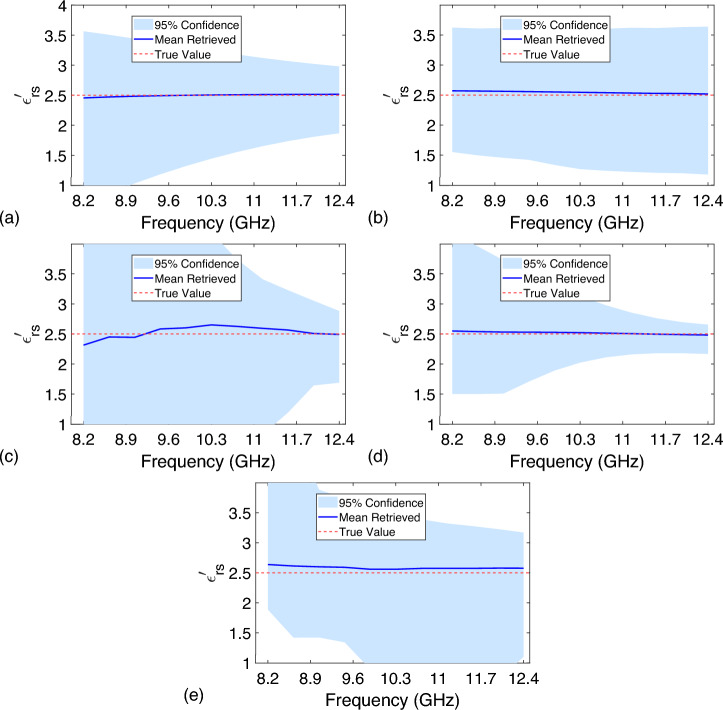
Extracted $\varepsilon _{rs}^{\prime }$ of the considered two-layer structure by (**a**) the Yang-Ma method in the study^32^, (**b**) the Hasar-1 method in the study^27^, (**c**) the Hasar et al. (2) method in the study^35^, (**d**) the Williams et al. method in the study^23^, and (**e**) the proposed method when *+5%* offset was introduced to the substrate permittivity ($\varepsilon _{rp}$).

**Fig. 13:**
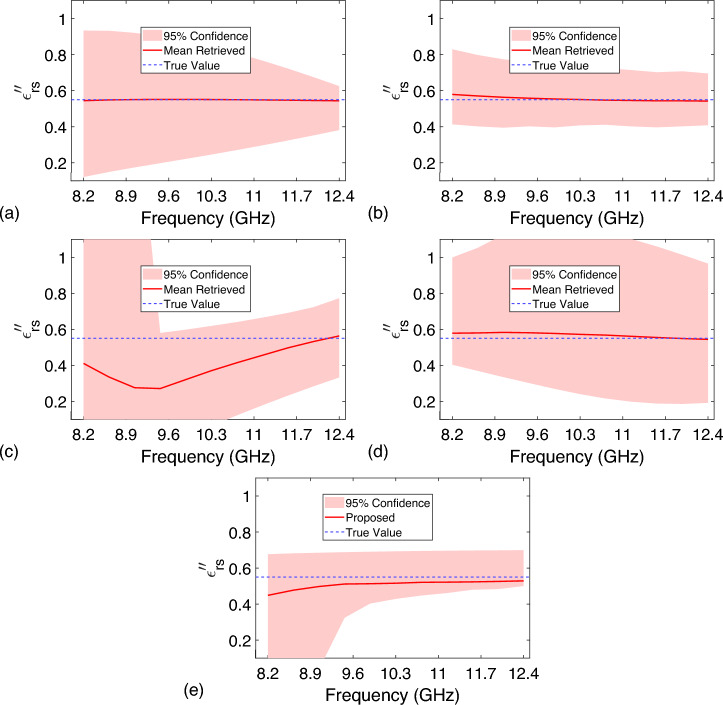
Extracted $\varepsilon _{rs}^{\prime \prime }$ of the considered two-layer structure by (**a**) the Yang-Ma method in the study^32^, (**b**) the Hasar-1 method in the study^27^, (**c**) the Hasar et al. (2) method in the study^35^, (**d**) the Williams et al. method in the study^23^, and (**e**) the proposed method when *+5%* offset was introduced to the substrate permittivity ($\varepsilon _{rp}$).

**Fig. 14:**
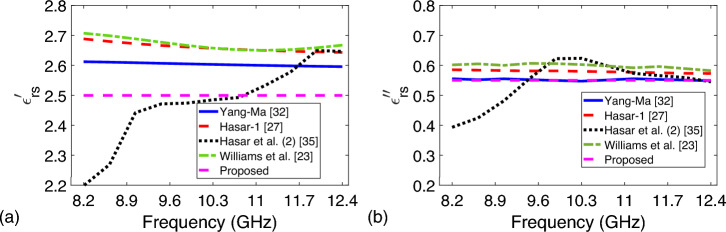
Extracted mean values of (**a**) $\varepsilon _{rs}^{\prime }$ and (**b**) $\varepsilon _{rs}^{\prime \prime }$ of the considered two-layer structure by the Yang-Ma method in the study^32^, the Hasar-1 method in the study^27^, the Hasar et al. (2) method in the study^35^, the Williams et al. method in the study^23^, and the proposed method when *+5%* offset was introduced to the substrate thickness ($L_{\text {sub}}$).

**Fig. 15:**
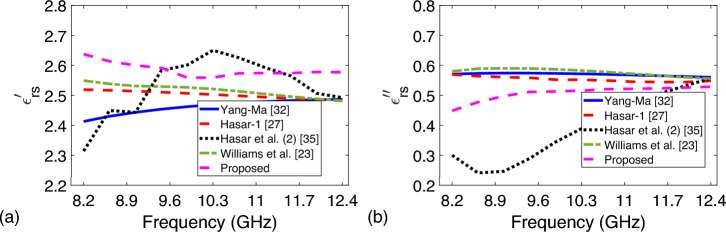
Extracted mean values of (**a**) $\varepsilon _{rs}^{\prime }$ and (**b**) $\varepsilon _{rs}^{\prime \prime }$ of the considered two-layer structure by the Yang-Ma method in the study^32^, the Hasar-1 method in the study^27^, the Hasar et al. (2) method in the study^35^, the Williams et al. method in the study^23^, and the proposed method when *+5%* offset was introduced to the substrate permittivity ($\varepsilon _{rp}$).

### Quantitative and uncertainty analyses

For high-frequency S-parameters measurements by a VNA, there are three distinct error types (systematic, random, and drift) that contribute to the budget of an overall uncertainty. Systematic errors entail directivity, source match, load match, isolation, tracking, etc. Random errors involve trace noise, connector repeatability, and cable flexure. As the final error type, drift errors are the long-term time-dependent errors caused by temperature changes and aging of internal components due to overuse of the VNA, which could be pacified by frequent re-calibration or a critical control over the measurement environment. In our present study, measurements were as much as shortened to eliminate the effect of the latter error type on measurements. Likewise, systematic errors in our measurement environment were corrected or removed by implementation of the TRL calibration procedure (validated by preliminary measurements after the calibration procedure). Besides, random errors can be quantified or mitigated by repeated measurements. Toward this goal, a quantitative analysis was performed using five consecutive independent measurements. Table 3 presents average and percentage variations of $\varepsilon _{rs}^{\prime }$ and $\varepsilon _{rs}^{\prime \prime }$ of the PVC sample determined for each case by different methods^23,29,30,32,34,35^ and our method. These variations can be considered as a metric for repeatability of measurements. It is seen from Table 3 that percentage variations of $\varepsilon _{rs}^{\prime }$ and $\varepsilon _{rs}^{\prime \prime }$ increase when precise $L_{\text {sam}}$ and/or $L_{\text {sub}}$ information is not available for the methods^23,29,30,32,34,35^. Additionally, improper information of reference-plane accompanies with an increased percentage variations of $\varepsilon _{rs}^{\prime }$ and $\varepsilon _{rs}^{\prime \prime }$ for the methods^23,29,32^, and the proposed method. On the other hand, percentage variations of $\varepsilon _{rs}^{\prime \prime }$ are greater than those of $\varepsilon _{rs}^{\prime }$ for any case for any tested method because the applied non-resonance type methods have limited accuracy for $\varepsilon _{rs}^{\prime \prime }$ of low-loss dielectric samples. Finally, considering uncertainties of S-parameters ($\Delta |S_{11}| = \Delta |S_{22}| \cong \Delta |S_{21}| = 0.01$ and $\Delta \theta _{11} = \Delta \theta _{22} \cong \Delta \theta _{21} = 1^o$), error bars were drawn at three different discrete frequencies (8.2 GHz, 10.3 GHz, and 12.4 GHz) for the proposed method as shown in Figs. 8, 9, and 10a.

**Table 3:** Average extracted $\varepsilon _{rs}^{\prime }$ and $\varepsilon _{rs}^{\prime \prime }$ along with their percentage variations of the PVC sample with $L_{\text {sam}} = 1.00$ mm at 10.3 GHz (mid-frequency) using five-independent measurements by the Williams et al. method in the study^23^, the Chao et al. method in the study^29^, the Hasar et al. (2) method in the study^35^, the Hasar-Bute method in the study^30^, the Yang-Ma method in the study^32^, the Hasar-Korkmaz method in the study^34^, and the proposed method.

		The Method						
	Case	^23^	^29^	^35^	^30^	^32^	^34^	Proposed
$\varepsilon _{rs}^{\prime }$	Case-I	*2.88 ± 0.07*	*2.85 ± 0.07*	*2.91 ± 0.07*	*2.89 ± 0.06*	*2.89 ± 0.08*	*2.90 ± 0.06*	*2.86 ± 0.05*
		(*± 2.50*%)	(*± 2.73*%)	(*± 2.61*%)	(*± 2.15*%)	(*± 2.80*%)	(*± 2.11*%)	(*± 2.03*%)
	Case-II	*2.92 ± 0.09*	*2.91 ± 0.09*	*2.95 ± 0.08*	*2.92 ± 0.08*	*2.92 ± 0.09*	*2.93 ± 0.09*	*2.86 ± 0.06*
		(*± 3.36*%)	(*± 3.17*%)	(*± 2.98*%)	(*± 2.78*%)	(*± 3.09*%)	(*± 3.11*%)	(*± 2.03*%)
	Case-III	*2.77 ± 0.08*	*2.76 ± 0.08*	*2.91 ± 0.07*	*2.78 ± 0.07*	*2.77 ± 0.08*	*2.78 ± 0.07*	*2.86 ± 0.05*
		(*± 3.03*%)	(*± 3.08*%)	(*± 2.61*%)	(*± 2.59*%)	(*± 3.03*%)	(*± 2.63*%)	(*± 2.03*%)
	Case-IV	*2.73 ± 0.07*	*2.79 ± 0.07*	*2.91 ± 0.07*	*2.89 ± 0.06*	*2.78 ± 0.08*	*2.90 ± 0.06*	*2.77 ± 0.08*
		(*± 2.86*%)	(*± 2.83*%)	(*± 2.61*%)	(*± 2.59*%)	(*± 2.88*%)	(*± 2.11*%)	(*± 2.93*%)
$\varepsilon _{rs}^{\prime \prime }$	Case-I	*0.12 ± 0.01*	*0.12 ± 0.01*	*0.13 ± 0.01*	*0.13 ± 0.01*	*0.12 ± 0.01*	*0.11 ± 0.01*	*0.11 ± 0.01*
		(*± 5.17*%)	(*± 4.88*%)	(*± 5.56*%)	(*± 5.60*%)	(*± 5.22*%)	(*± 5.40*%)	(*± 5.36*%)
	Case-II	*0.10 ± 0.01*	*0.11 ± 0.01*	*0.11 ± 0.01*	*0.11 ± 0.01*	*0.11 ± 0.01*	*0.11 ± 0.01*	*0.11 ± 0.01*
		(*± 4.95*%)	(*± 5.66*%)	(*± 5.45*%)	(*± 5.50*%)	(*± 4.67*%)	(*± 6.31*%)	(*± 5.36*%)
	Case-III	*0.12 ± 0.01*	*0.13 ± 0.01*	*0.13 ± 0.01*	*0.13 ± 0.01*	*0.12 ± 0.01*	*0.12 ± 0.01*	*0.11 ± 0.01*
		(*± 4.88*%)	(*± 5.43*%)	(*± 5.56*%)	(*± 5.34*%)	(*± 4.84*%)	(*± 5.93*%)	(*± 5.36*%)
	Case-IV	*0.13 ± 0.01*	*0.12 ± 0.01*	*0.13 ± 0.01*	*0.13 ± 0.01*	*0.12 ± 0.01*	*0.11 ± 0.01*	*0.12 ± 0.01*
		(*± 4.80*%)	(*± 5.65*%)	(*± 5.56*%)	(*± 5.60*%)	(*± 4.96*%)	(*± 5.40*%)	(*± 5.78*%)

To determine whether the deviations observed among the different extraction methods (detailed in Subsects. 3.4 to 3.6) are meaningful or merely artifacts of measurement noise, we compared the method-induced errors against these established measurement uncertainties. As shown in Table 3, the baseline random variation (measurement uncertainty) for the proposed method under ideal conditions (Case-I) is approximately 2.03% for $\epsilon _{rs}^{\prime }$ and 5.36% for $\epsilon _{rs}^{\prime \prime }$. In contrast, when the competing methods are subjected to artificial offsets in $L_{\text {sub}}$ or $L_{\text {sam}}$ (Cases II, III, and IV), the resulting deviations drastically exceed these baseline limits. For example, a +5% offset in $L_{\text {sam}}$ introduces a change in $\epsilon _{rs}^{\prime }$ of approximately 8.1% for the Chao et al. method. Because these method-induced percentage variations are significantly larger than our established baseline uncertainty limits, we conclude that the performance differences observed among the methods are physically meaningful and fall well outside the margin of standard measurement noise.

Finally, an uncertainty analysis was performed to evaluate the effect of the substrate parameters on extracted $\varepsilon _{rs}$ by various methods. Toward this goal, the parameters utilized in Subsect. 2.3 were re-used here ($\varepsilon _{rs} = 2.50 - j 0.55$, $\varepsilon _{rp} = 6.0 - j 0.45$, $L_{\text {sam}} = 1.00$ mm, $L_{\text {sub}} = 2.84$ mm, and $f_c = 6.557$ GHz). An uncertainty analysis based on the Monte Carlo method was implemented since traditional the law of propagation of uncertainty is not much feasible for non-linear models. Here, the number of realizations were set to 500, uncertainty in substrate thickness or substrate permittivity was arranged to 5%, and confidence level was considered to be 95%. Figs. 10, 11, 12 and 13 illustrate the spectral dependencies of $\varepsilon _{rs}^{\prime }$ and $\varepsilon _{rs}^{\prime \prime }$ of the considered two-layer structure extracted by the Yang-Ma method in the study^32^, the Hasar-1 method in the study^27^, the Hasar et al. (2) method in the study^35^, the Williams et al. method in the study^23^, and the proposed method when *+5%* offset was introduced to the substrate thickness ($L_{\text {sub}}$) and when *+5%* offset was introduced to the substrate permittivity ($\varepsilon _{rp}$), respectively. In addition, Figs. 14 and 15 present the mean $\varepsilon _{rs}^{\prime }$ and (b) $\varepsilon _{rs}^{\prime \prime }$ values of the considered two-layer structure by the Yang-Ma method in the study^32^, the Hasar-1 method in the study^27^, the Hasar et al. (2) method in the study^35^, the Williams et al. method in the study^23^, and the proposed method when *+5%* offset was introduced to the substrate thickness ($L_{\text {sub}}$) and when *+5%* offset was introduced to the substrate permittivity ($\varepsilon _{rp}$). These figures could be considered as the error the each tested extraction may introduce in evaluation of the true $\varepsilon _{rs} = 2.50 - j 0.55$ value.

The following points are noted from the results in Figs. 10, 11, 12, 13, 14 and 15. First, while extracted $\varepsilon _{rs}^{\prime }$ and $\varepsilon _{rs}^{\prime \prime }$ by the proposed method does not change with the *+5%* offset in $L_{\text {sub}}$, those retrieved by the Yang-Ma method in the study^32^, the Hasar-1 method in the study^27^, the Hasar et al. (2) method in the study^35^, and the Williams et al. method in the study^23^ are influenced this offset. This is because our proposed method is free-from $L_{\text {sub}}$ information whereas the Yang-Ma method in the study^32^, the Hasar-1 method in the study^27^, the Hasar et al. (2) method in the study^35^, and the Williams et al. method in the study^23^ all rely on this information in the extraction procedure. Second, the Yang-Ma method in the study^32^, the Hasar-1 method in the study^27^, the Hasar et al. (2) method in the study^35^, the Williams et al. method in the study^23^, and our proposed method are all affected by the *+5%* offset in $\varepsilon _{rp}$. This is because they all necessitate accurate information of $\varepsilon _{rp}$ in the determination of $\varepsilon _{rs}$. Third, the Hasar et al. (2) method in the study^35^ is influenced by the $L_{\text {sub}}$ or $\varepsilon _{rp}$ offset more than the Yang-Ma method in the study^32^, the Hasar-1 method in the study^27^, the Williams et al. method in the study^23^, and the proposed method. The reason for this could be the need for additional measurements (sample-substrate S-parameter measurements in addition to substrate-only measurements) in comparison with the measurements needed by the Yang-Ma method in the study^32^, the Hasar-1 method in the study^27^, the Williams et al. method in the study^23^, and the proposed method.

**Fig. 16:**
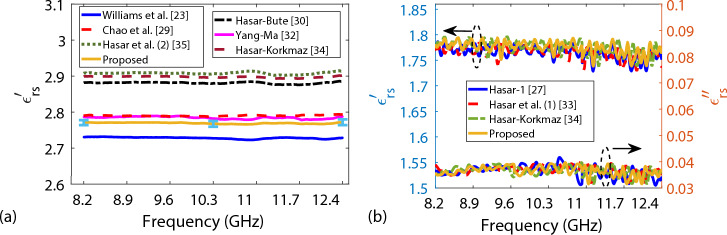
(**a**) Extracted $\varepsilon _{rs}^{\prime }$ of the PVC sample with $L_{\text {sam}} = 1.00$ mm for the Case-IV (unknown reference-plane information) by the Williams et al. method in the study^23^, the Chao et al. method in the study^29^, the Hasar et al. (2) method in the study^35^, the Hasar-Bute method in the study^30^, the Yang-Ma method in the study^32^, the Hasar-Korkmaz method in the study^34^, and the proposed method [Error bars for the extracted parameters by the proposed method are given at discrete frequencies 8.2 GHz (minimum frequency), 10.3 GHz (mid-frequency), and 12.4 GHz (maximum frequency)] and (b) retrieved $\varepsilon _{rs}^{\prime }$ and $\varepsilon _{rs}^{\prime \prime }$ of the zinc oxide thin film extracted by the Hasar-1 method in the study^27^, the Hasar et al. (1) method in the study^33^, the Hasar-Korkmaz method^34^, and the proposed method.

### Thin film characterization

In order to test the applicability and performance of our proposed method, $\varepsilon _{rs}$ of a thin film was retrieved^34^. This film (zinc oxide from zinc nitrate hexahydrate) with $L_{\text {sam}} \cong 1.0 \mu$m was deposited on a fused quartz substrate ($L_{\text {sub}} \cong 2.05 \mp 0.063$ mm measured by the high precision caliper from Mitutoyo) around $400^o$C at 0.1 M ratio using the spray pyrolysis technique. The permittivity of the substrate $\varepsilon _{rp}$ was taken as *≅ 3.78 (1 - j 0.00025)* using the reference data^53^. Thereafter, it was annealed at $550^o$C for 2 hours^54^. Figure 16a and b show, respectively, $\varepsilon _{rs}^{\prime }$ and $\varepsilon _{rs}^{\prime \prime }$ of this thin film extracted by the Hasar-1 method in the study^27^, the Hasar et al. (1) method in the study^33^, the Hasar-Korkmaz method^34^, and the proposed method. It is noted from the results in Fig. 16b that extracted $\varepsilon _{rs}^{\prime }$ (and $\varepsilon _{rs}^{\prime \prime }$) by the Hasar-1 method in the study^27^, the Hasar et al. (1) method in the study^33^, the Hasar-Korkmaz method^34^, and the proposed method are all in good agreement with each other over the entire frequency band. This validates the applicability of our method for thinner samples. Nonetheless, it is noteworthy that even though such an agreement is established by different extraction methods, extracted relative permittivity values in Fig. 16b are partly lower than those typically reported for dense bulk ZnO (around 7.0) in the literature^55^. Such a discrepancy is attributed to the porous microstructure of the thin film, high-frequency dielectric dispersion in the microwave regime, and thin-film size effects that suppress polarization mechanisms. Effective medium considerations indicate that moderate porosity can significantly reduce the effective dielectric constant toward values close to unity. The physical validity of the extracted parameters was verified through forward electromagnetic simulations and consistency checks between measured and reconstructed S-parameters, confirming that the obtained values are not numerical artifacts.

### Advantages and limitations of our proposed extraction method

Our proposed method evaluates $\varepsilon _{rs}$ of thinner samples without resorting to information of sample thickness and substrate thickness utilizing a new mathematical model (the state-space approach) leading to explicit expressions free-from implementation of any numerical method. Although our proposed method and the methods in the studies^1,9,23,27–36^ attack conceptually the same problem (characterization of relative permittivity of the sample on a substrate material), our method is unique in the sense that it has a different mathematical formulation or analysis rendering that it does not require information of both the sample thickness and the substrate thickness while characterizing the relative permittivity of the sample. This formulation takes advantage of considering transmission measurements, involving the information of sample thickness and the substrate thickness, in addition to reflection measurements, as different from the methods in the studies^33,34^ which require one-port reflection-only measurements. It is noteworthy from (1)-(45) that while the $g_{\text {II}}$ parameter (associated with the sample wave impedance) is a function of $\varepsilon _{rs}$ only, the $e^{\lambda _1^{\text {(II)}}}$ parameter (linked with propagation factor inside the sample) [$e^{\lambda _3^{\text {(II)}}} = e^{-\lambda _1^{\text {(II)}}}$] varies with both $\varepsilon _{rs}$ and $L_{\text {sam}}$. Our proposed method first eliminates the dependence of $\Phi _{22}^{(\text {T})}$, $\Phi _{23}^{(\text {T})}$, $\Phi _{32}^{(\text {T})}$, and $\Phi _{33}^{(\text {T})}$ (which are all functions of measured S-parameters) on the $e^{\lambda _1^{\text {(II)}}}$ parameter through $\Omega _1$ and $\Omega _2$ quantities from (35)-(40) and then extracts $\varepsilon _{rs}$ relying on the $g_{\text {II}}$ parameter only from (43) and (44). Thereby, this categorizes our proposed method into an extraction method free-from the sample thickness and the substrate thickness.

Our method, however, necessitates information about the precise location of the sample within its measurement cell (Fig. 16a). For applications where accurate reference-plane information of the sample (and substrate) is not available, the methods in the studies^30,34,35^ could be preferred. Nevertheless, the reference-plane dependence of our proposed method can be partly mitigated by flushing the sample over the substrate with either the left or right terminal of the measurement cell. Then, our proposed method could be employed for $\varepsilon _{rs}$ determination of samples without resorting sample thickness and substrate thickness information, especially useful for i) the characterization of radar-absorbent material (RAM) coatings involving curved or complex surfaces in stealth technology and ii) measuring the permittivity of anti-corrosive paints or thermal barrier coatings on turbine blades. In addition, although our method has relatively high accuracy for medium and/or low-loss dielectric samples, its accuracy decreases for very low-loss dielectric materials which have considerably lower loss tangent values (tan$\delta = \varepsilon _{rs}^{\prime \prime } / \varepsilon _{rs}^{\prime }$). This is a common drawback of non-resonance type extraction methods^1–3,10–12,27–37^. As the opposite case, if the loss inside the sample increases due to higher tan*δ* values, the level of measured $|S_{21}^{\text {(T)}}|$ could be lower than the noise level of the VNA instrument so that noise present in the measurement system starts to play a dominant role in the extraction procedure, leading to a reduction in the accuracy of $\varepsilon _{rs}$ determination by the proposed method. Finally, our proposed method utilizes reflection and transmission S-parameters of one orientation of the sample over the substrate material in Fig. 1 (not different orientations) for a given electric and magnetic field pattern of the dominant mode only (one electromagnetic pattern), our proposed method in its present form is not applicable for $\varepsilon _{rs}$ determination of anisotropic samples.

## Conclusion

An extraction method is proposed for accurate $\varepsilon _{rs}$ determination of thinner dielectric low-loss samples positioned/deposited on a substrate material from waveguide non-resonance type two-port S-parameter measurements. It does not necessitate sample and substrate thickness information in its execution. The theoretical model established over the state transition matrix along with state vector is unique in the sense that this model has been implemented for the first time for a two-layer composite structure to determine a closed-form expression for $\varepsilon _{rs}$ linking measured S-parameters, thus eliminating the need for a numerical analysis or tool required by previous studies. After validating and evaluating its accuracy against noise and any potential air gap between the sample and its substrate material, X-band waveguide measurements of the thinner (1.00 mm) PVC sample for four different scenarios and of a thin film with a thickness on the order of 1*μ*m were carried out. Different one-port and two-port non-resonance type measurements were applied for assessing the performance of our proposed method. From these measurements, it is noted that while our proposed method does not require precise sample and/or substrate thickness information, other extraction methods require, thus manifesting the importance of this information while evaluating $\varepsilon _{rs}$. Furthermore, imprecise sample and/or substrate thickness information introduces additional percentage variations in $\varepsilon _{rs}$ determination. Our proposed method can be applied especially for $\varepsilon _{rs}$ determination of thin-film, radar-absorbent material coatings, anti-corrosive paints, or thermal barrier coatings where a change in sample thickness (and substrate thickness) is intolerable from one fabrication to another.

## Data Availability

The datasets used and analyzed during the current study are available from the corresponding author (U.C.H) upon reasonable request.
